# Sino-European Transcontinental Basic and Clinical High-Tech Acupuncture Studies—Part 3: Violet Laser Stimulation in Anesthetized Rats

**DOI:** 10.1155/2012/402590

**Published:** 2012-04-18

**Authors:** Xin-Yan Gao, Gerhard Litscher, Kun Liu, Bing Zhu

**Affiliations:** ^1^Stronach Research Unit for Complementary and Integrative Laser Medicine, Research Unit of Biomedical Engineering in Anesthesia and Intensive Care Medicine, TCM Research Center Graz, Medical University of Graz, Auenbruggerplatz 29, 8036 Graz, Austria; ^2^Department of Physiology, Institute of Acupuncture and Moxibustion, China Academy of Chinese Medical Sciences, Dongzhimen Nanxiaojie Street, Beijing 100700, China

## Abstract

The aim of this study was to determine the effect of violet laser stimulation on three acupuncture points in anesthetized rats and to test the hypothesis that violet laser light can modulate neurovegetative parameters like heart rate (HR), heart rate variability (HRV), and mean arterial blood pressure (MAP). Recordings were performed in 10 male anesthetized rats under three conditions in Beijing, and monitored with equipment from Graz, where also data analysis was performed. For stimulation a violet laser (emitted wavelength 405 nm, laser output 1 mW, continuous mode) was used. The electrocardiograms were recorded by an HRV Medilog AR12 system during laser acupuncture stimulation of the head, ear, and body (Baihui, “heart” ear acupoint, Zusanli). HR changed significantly only during (*P* = 0.013) and after (*P* = 0.038) stimulation at Baihui. Total HRV and the low frequency/high frequency ratio showed insignificant changes. There was an insignificant decrease in MAP after stimulation of Baihui acupoint. Violet laser stimulation offers a method to induce acute effects in HR and HRV in rats. Although the precise mechanism of this effect remains to be determined, alterations are significant. Violet laser stimulation on the Baihui acupoint could readily be translated to clinical studies.

## 1. Introduction

Basic and clinical applications of low-power laser stimulation are numerous. The field of research is characterized by a variety of different methodologies and uses of various light sources with different parameters (wavelength, output power, continuous wave or pulsed operation modes, and pulse parameters). Although in recent years longer wavelengths (650 to 900 nm, that is, red and infrared) and higher output powers (up to 150 mW) have been preferred in medical therapeutic devices, ultra-low-level laser stimulation is still a topic of animal experimental and human research [1, 2].

In the present study, we used for the first time ultra-low-level laser stimulation (405 nm; 1 mW; continuous mode) in anesthetized rats under stable conditions and analyzed the effects on physiological neurovegetative parameters. Similar to our first study in this series [3] the data were recorded for 10 rats in Beijing, China, and the data analysis was performed in Graz, Austria. A system normally used for human data analysis has been specifically adapted in Europe for these studies in rats [3].

## 2. Animals and Methods

### 2.1. Sprague-Dawley Rats and Blood Pressure Monitoring

Ten male Sprague-Dawley rats were kept in an animal house maintained at 21 ± 2°C with a 12-hour light-dark cycle and were given free access to food and water. The weight of the rats was 300–350 g. The animals were initially anesthetized with an intraperitoneal injection of 10% urethane (1.0 g/kg, Sigma-Aldrich, St. Louis, USA).

The left common carotid artery was cannulated with a polyethylene catheter filled with physiological saline containing heparin (200 IU/mL, Sigma-Aldrich, St. Louis, USA) to record mean arterial pressure (MAP) via a blood pressure transducer (DA100, Biopac Systems, Inc., Aero Camino Goleta, USA) and amplifier (MP150, Biopac Systems, Inc., Aero Camino Goleta, USA). This signal was registered on Micro1401 and Spike2 (CED, Cambridge Electronic Design Limited, Cambridge, UK) data acquisition unit and software. The procedure was the same as in our first study of this series [3]. The depth of anesthesia was monitored by changes in MAP, and additional anesthetic (urethane 0.3 g/kg) was given if the animal showed large fluctuations in baseline AP or a withdrawal response to a pinch of the paw. After tracheal cannulation, the animals breathed spontaneously, and their core temperature was maintained at 37.0 ± 0.5°C by a feedback-controlled electric blanket (FHC Inc., Bowdoin, USA). The animals were sacrificed after the investigation by an overdose of anesthetics. The experiments were conducted in accordance with the *Guide for Care and Use of Laboratory Animals* issued by the National Institutes of Health (China), and the procedures were approved by the Institutional Animal Care and Use Committee of the China Academy of Chinese Medical Sciences.

### 2.2. Electrocardiographic Monitoring in Rats

The data from electrocardiograms (ECGs) were recorded by an HRV Medilog AR12 (Huntleigh Healthcare, Cardiff, UK, and Leupamed GmbH, Graz, Austria) system. The data were analyzed using specially adapted software (Huntleigh Healthcare) [3]. The sampling rate of the recorder is 4096 Hz. All raw data from the rat experiments were stored digitally on a 32 MB compact flash memory card. After removing the card from the portable system in the lab of the Institute of Acupuncture and Moxibustion at the China Academy of Chinese Medical Sciences in Beijing, the data were read by an appropriate card reader connected to a standard computer and sent to the lab at the Stronach Research Unit for Complementary and Integrative Laser Medicine in Graz.

As described in previous publications, HRV is measured as a percent change in sequential chamber complexes called RR-intervals in the ECG. It can be quantified in the time domain and in the frequency range by analyzing the ECG power spectra [3–9]. The task force of the European Society of Cardiology and the North American Society of Pacing and Electrophysiology recommended the HRV parameters already in 1996 [10]. The mean HR, total HRV, and LF (low frequency)/HF (high frequency) ratio of the HRV were evaluated [10].

### 2.3. Violet Laser Stimulation and Procedure

Three locations were selected for laser stimulation. All points can regulate cardiovascular and neurovegetative functions [11–13]. The points were identified by anatomical marks and previous reports [11–13]. Baihui (GV20) is located at the continuation of the line connecting the highest points of the ear, on the median line of the head. The “heart” ear acupoint is located at the inferior concha. Zusanli (ST36) is located on the anterolateral side of the hindlimb near the anterior crest of the tibia below the knee under the tibialis anterior muscle [11–13].

**Figure 1:**
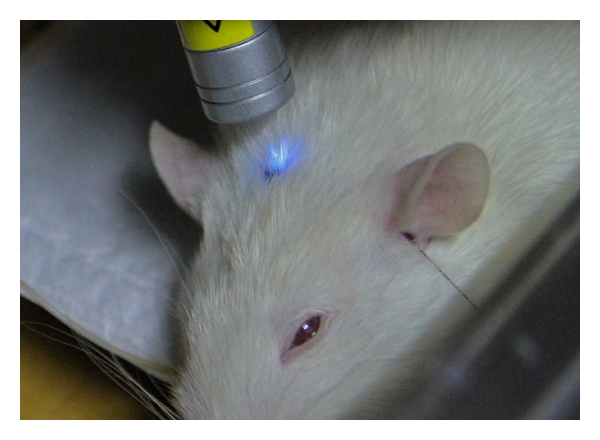
Violet laser stimulation in a rat at the Baihui acupuncture point.

**Figure 2:**
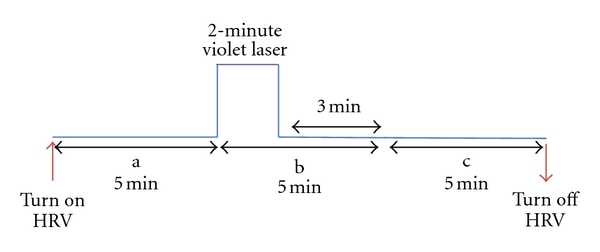
Experimental procedure for violet laser stimulation (405 nm) at the three acupoints.

For laser stimulation, an instrument (Conrad Electronic SE, Hirschau, Germany) with a wavelength of 405 nm (violet) and an output power of 1 mW with a continuous beam was used for a duration of 2 min (Figure 1). The time scale of each stimulation is shown in Figure 2. The order of point stimulation was randomized, and the time between the investigations of the different acupoints was at least 10 minutes.

The measurement profile and measurement sessions (a–c) are shown in Figure 2. Three measurement periods were compared: one before stimulation (a); one immediately after the beginning of the 2-minute violet laser acupuncture stimulation (b); one as a second control (c). This scheme was also used, in an adapted version, in a previous investigation in rats (part 1 of this series, [3]).

### 2.4. Statistical Analysis

The data were analyzed using one-way repeated measures analysis of variance (ANOVA) (SigmaPlot 11.0, Systat Software Inc., Chicago, USA). Post hoc analysis was performed using Holm-Sidak test. The level of significance was defined as *P* < 0.05.

## 3. Results

**Figure 3:**
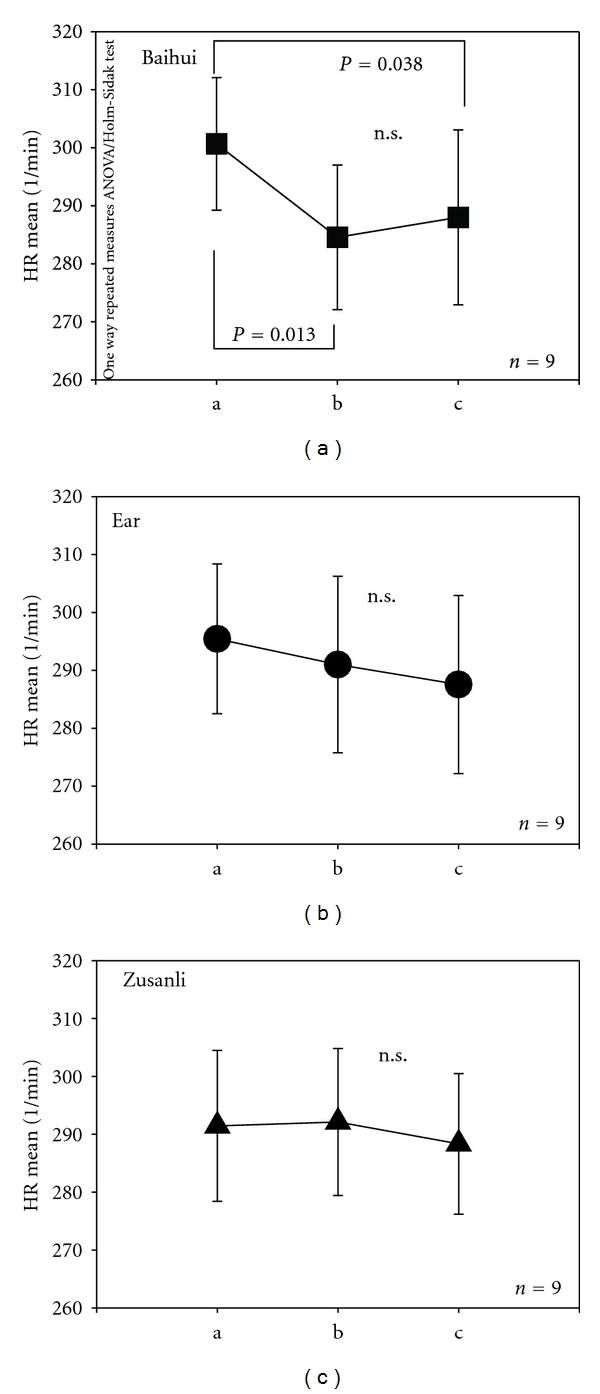
Diagrams displaying the mean heart rate (HR mean) and standard error of the mean (SE) of the 9 rats. There is a significant decrease of HR mean during (b) and after (c) violet laser stimulation at Baihui compared to the reference interval before stimulation (a). The different measurement phases (a–c; compare with Figure 2) and acupuncture points (Baihui; ear acupuncture: heart point; Zusanli) are indicated.

**Figure 4:**
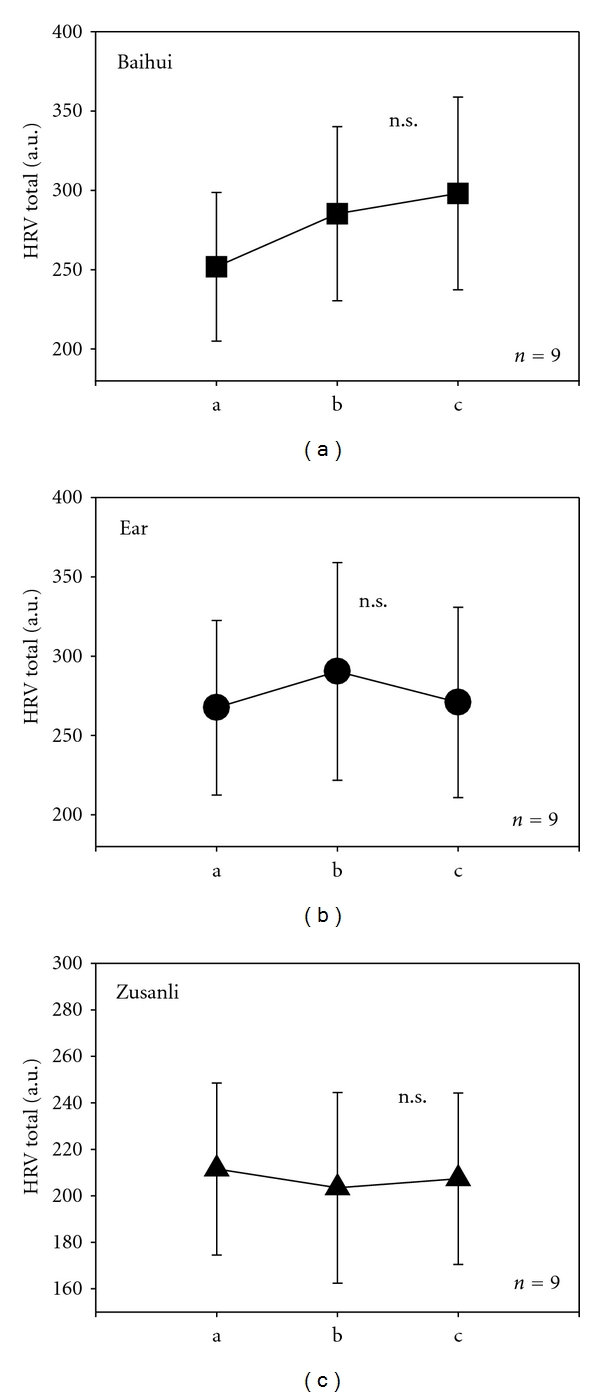
Graphical plots displaying total heart rate variability (HRV total) for the 9 rats. Note the marked but insignificant increase in HRV total after violet laser stimulation of the acupuncture point Baihui (a). For further explanation, compare with Figure 3.

Data analysis was performed successfully in 9 of the 10 rats. In one rat, mean HR in the control intervals was lower than 200/min and thus not included in the analysis. Figures 3 and 4 show the mean HR and HRV total (total heart rate variability) from the ECG recordings from 9 of the 10 rats during the three measurement phases (a, b, and c) as well as before, during, and after stimulation at the Baihui acupoint (a). The results from the stimulation of the ear and body point, respectively, are also shown ((b) ear; (c) Zusanli). There was a significant change in HR during (*P* = 0.013) and after (*P* = 0.038) the stimulation only when the Baihui acupoint was stimulated (Figure 3(a); compare also with Figure 1). 

HRV total increased insignificantly (n.s.) during violet acupuncture stimulation at the acupoint Baihui and the ear acupoint (Figure 4). However, during stimulation of Zusanli, it decreased insignificantly. At the reference interval at the end of the measurement, there was an increase in HRV total only after stimulation at Baihui (Figure 4(a)).

**Figure 5:**
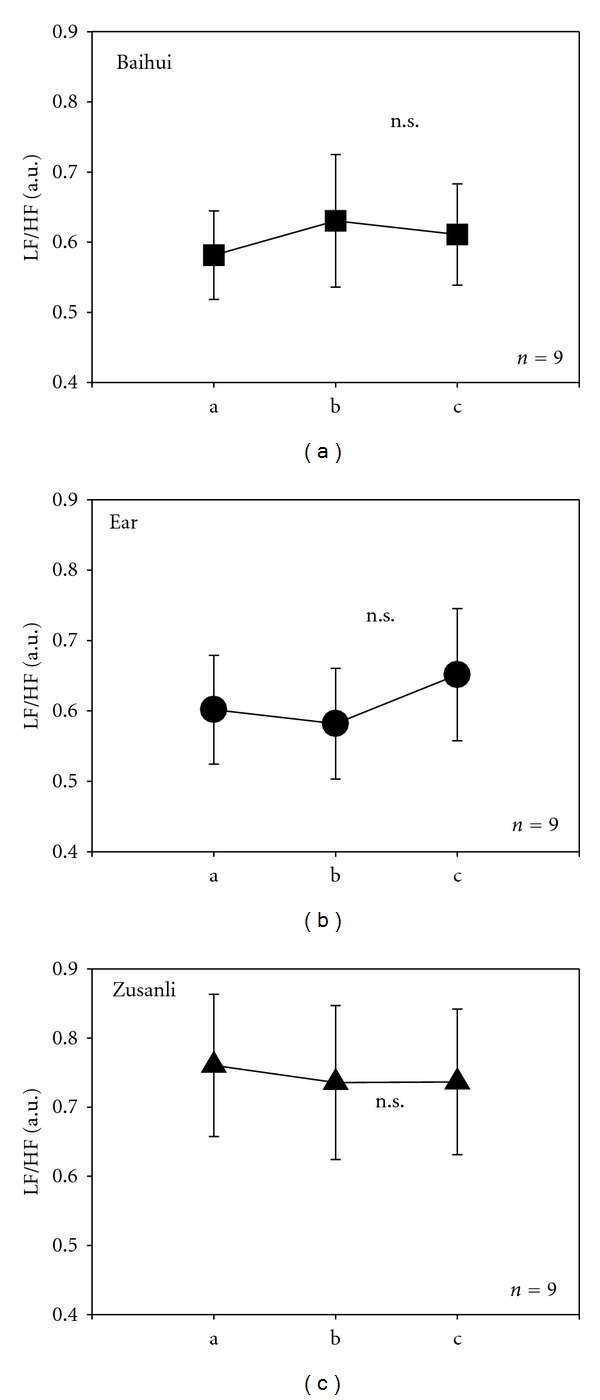
LF (low-frequency)/HF (high-frequency) ratio. For further explanation, see Figures 3 and 4.

Furthermore, continuous HR-HRV monitoring showed insignificant alterations in the LF/HF ratio after acupuncture stimulation at the three points in rats (Figure 5).

**Figure 6:**
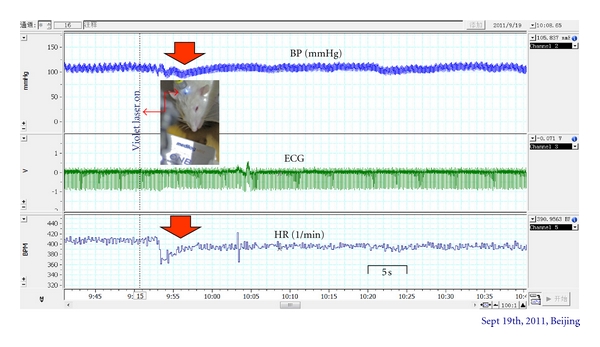
Original monitoring protocol of blood pressure (BP), raw electrocardiographic signal (ECG), and heart rate (HR) before and immediately after onset of violet laser stimulation. Note the decrease in BP and HR (red arrow).

**Figure 7:**
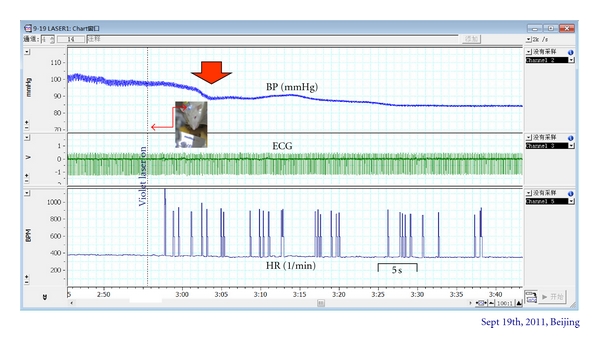
Original monitoring protocol of blood pressure (BP), raw electrocardiographic signal (ECG), and heart rate (HR) before and immediately after onset of violet laser stimulation.

Figures 6 and 7 show computer chart records of typical experiments. Changes in blood pressure (BP), ECG, and HR after stimulation with violet laser at Baihui are demonstrated. Short-term decreases of BP and HR are shown in Figure 6 (“on effect”).

In Figure 7, the continuous decrease of BP after violet laser stimulation onset is documented.

**Figure 8:**
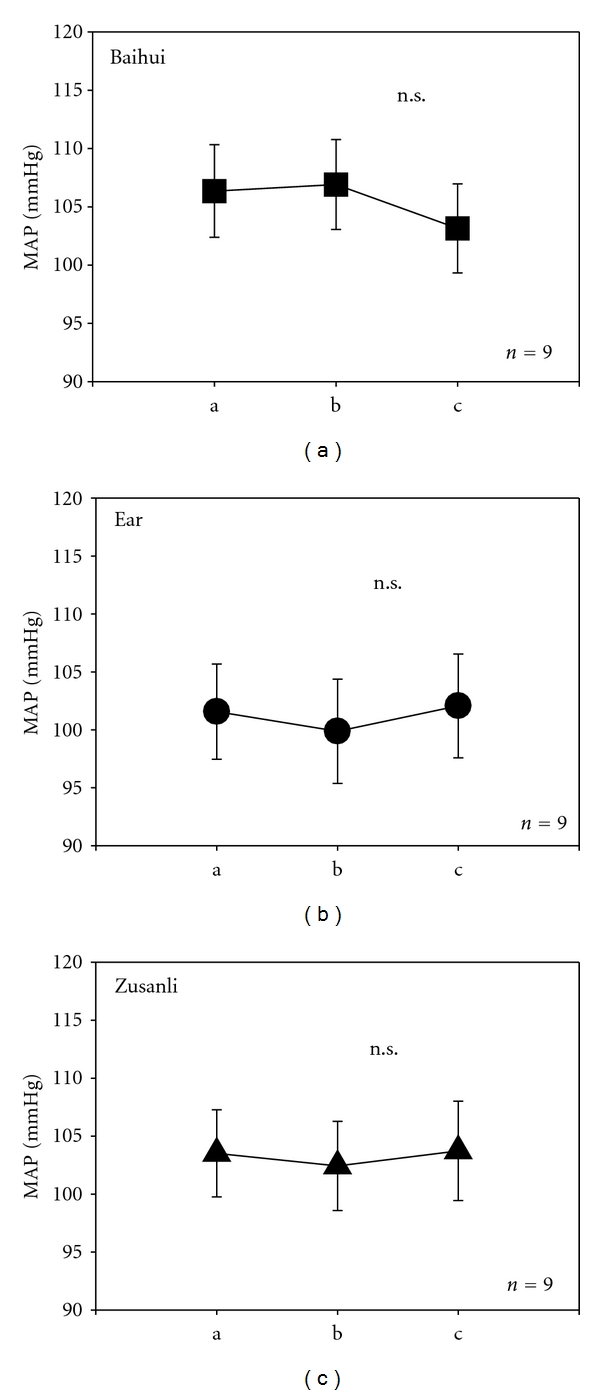
Diagrams showing changes in mean arterial pressure (MAP) for the three different stimulation areas using violet laser.

The data of the MAP of all 9 rats are summarized in Figure 8. Note the insignificant (n.s.) decrease of MAP in the control phase after stimulation with violet laser at Baihui.

## 4. Discussion

Laser light is a good alternative to metal needles for stimulation of acupuncture points, and it has been used successfully for several decades. However, to date there are only few studies proving the effectiveness of this kind of acupuncture stimulation. Most publications focus on red or infrared laser stimulation, and there are several relevant studies [14–20].

Violet laser acupuncture using a wavelength of 405 nm has been investigated in only a few scientific studies performed in humans by the research group in Graz [21–25]. To the best of our knowledge, it has not yet been used in animal experimental studies on acupuncture. However, some laboratory and clinical studies over the past ten years have shown that low-level laser stimulation using wavelengths of 633 or 670 nm and extremely low power densities (about 0.15 mW/cm²) is capable of eliciting significant biological effects [2].

In the present study, we used such a low-level violet laser stimulation (405 nm; <1 mW) for acupuncture research in rats for the first time. In previous studies in rats, it could be demonstrated that photobiomodulation using light with an 810 nm and 150 mW diode laser can be used as noninvasive treatment for acute spinal cord injury, potentially acting through an immunomodulatory mechanism. This suggests that light will be a useful treatment for humans [26]. Beside application at the spinal cord, it has also been shown that pulsed infrared light alters neural activity in the rat somatosensory cortex *in vivo* [27]. In this study, infrared neural stimulation was found to evoke an intrinsic response of similar magnitude to that induced by tactile stimulation. The authors conclude that infrared light can be used safely and effectively to manipulate neural firing.

In contrast to the previous findings in humans at the Medical University in Graz, we could demonstrate in this animal experimental study that ultra-low-level violet laser stimulation (≤1 mW) can modulate physiological and neurovegetative parameters after stimulating the Baihui acupuncture point. In the present study, there was also a clear on/off-effect when the laser was activated/deactivated (see typical examples in Figures 6 and 7). A similar effect with violet laser was shown in humans by Litscher et al. [21]. In that study, violet laser acupuncture (405 nm; 110 mW) at the acupoint Dazhui (GV14; on the same meridian as Baihui (GV20) in the present study) also induced an on/off-effect, but in different parameters, namely, the blood flow velocities in the basilar and middle cerebral arteries in the brain. In our present study, a more than 100 times lower output power was used in rats. Although the scalp bone of a rat is much thinner than that of a human subject, we suppose that there was no intensive direct radiation of the brain using the violet laser due to the intact skull of the rats. The on/off-effect of the violet laser stimulation could possibly be explained by the open eye of the rat; however, in human subjects, this explanation could be excluded. Of course, it would be possible to control this in future animal experimental studies.

Acupuncture stimulation at Baihui has also been investigated in other studies in rats. For example, Chuang et al. [28] stated that Baihui stimulation reduced cerebral infarct and increased dopamine levels in chronic cerebral hypoperfusion and ischemia-reperfusion injured Sprague-Dawley rats. Regular stimulation over a period of four weeks enhanced cognition and memory function of the rats.

The use of laser light as complementary or alternative method to acupuncture needle stimulation has been promoted for some decades. However, there are only few systematic assessments of evidence to support the effectiveness of this modern technical application of acupuncture to date [29].

In several human studies, Litscher et al. [21–25] have shown that violet laser acupuncture can yield reproducible effects. These previous results were confirmed by the results of the present animal experimental study.

## 5. Conclusions

The following conclusions can be drawn from the results of this second animal experimental transcontinental study:

- heart rate changes significantly during ultra-low-level violet laser stimulation of Baihui in anesthetized rats;
- total HRV changes insignificantly during violet laser application at Baihui, “heart” ear point, and Zusanli. However, there was a trend towards an increase in HRV total during and after stimulation of Baihui;
- the LF/HF ratio showed no significant changes;
- mean arterial pressure decreased (markedly, yet insignificantly) after violet laser stimulation of Baihui in rats.

## References

[1] Vo-Dinh T (2003). *Biomedical Photonics Handbook*.

[2] Baratto L, Calzà L, Capra R (2011). Ultra-low-level laser therapy. *Lasers in Medical Science*.

[3] Gao XY, Liu K, Zhu B, Litscher G (2012). Sino-European transcontinental basic and clinical high-tech acupuncture studies, part 1: auricular acupuncture increases heart rate variability in anesthetized rats. *Evidence-Based Complementary and Alternative Medicine*.

[4] Litscher G (2010). Transcontinental and translational high-tech acupuncture research using computer-based heart rate and " Fire of Life" heart rate variability analysis. *Journal of Acupuncture and Meridian Studies*.

[5] Litscher G, Wang L, Valentini J (2011). Biomedical teleacupuncture between China and Austria using heart rate variability, Part 1: poststroke patients. *Evidence-Based Complementary and Alternative Medicine*.

[6] Gao X-Y, Wang L, Gaischek I, Michenthaler Y, Zhu B, Litscher G (2012). Brain-modulated effects of auricular acupressure on the regulation of autonomic function in healthy volunteers. *Evidence-Based Complementary and Alternative Medicine*.

[7] Litscher G (2007). Bioengineering assessment of acupuncture, Part 7: heart rate variability. *Critical Reviews in Biomedical Engineering*.

[8] Litscher G (2009). Modernization of traditional acupuncture using multimodal computer-based high-tech methods-recent results of blue laser and teleacupuncture from the medical university of Graz. *Journal of Acupuncture and Meridian Studies*.

[9] Litscher G, Litscher D (2010). ‘Fire of Life’ analysis of heart rate variability during alpine skiing in Austria. *North American Journal of Medical Sciences*.

[10] Malik M, Camm AJ, Bigger Jr. JT (1996). Heart rate variability. Standards of measurement, physiological interpretation, and clinical use. *European Heart Journal*.

[11] Gao XY, Zhang SP, Zhu B, Zhang HQ (2008). Investigation of specificity of auricular acupuncture points in regulation of autonomic function in anesthetized rats. *Autonomic Neuroscience*.

[12] Gao XY, Li YH, Liu K (2011). Acupuncture-like stimulation at auricular point Heart evokes cardiovascular inhibition via activating the cardiac-related neurons in the nucleus tractus solitarius. *Brain Research*.

[13] Guo L, Xu JF, Liu J (2010). Electroacupuncture, calpain I expression, and survival of hippocampal neurons in cerebral ischemia reperfusion rats. *Medical Acupuncture*.

[14] Litscher G (2008). High-tech laser acupuncture is Chinese medicine. *Medical Acupuncture*.

[15] Litscher G, Schikora D (2005). *Laserneedle-Acupuncture. Science and Practice*.

[16] Litscher G (2009). Ten years evidence-based high-tech acupuncture—a short review of peripherally measured effects. *Evidence-Based Complementary and Alternative Medicine*.

[17] Litscher G (2009). Ten years evidence-based high-tech acupuncture—a short review of centrally measured effects. *Evidence-Based Complementary and Alternative Medicine*.

[18] Litscher G (2010). Ten years evidence-based high-tech acupuncture part 3: a short review of animal experiments. *Evidence-Based Complementary and Alternative Medicine*.

[19] Litscher G (2006). Electroencephalogram-entropy and acupuncture. *Anesthesia and Analgesia*.

[20] Litscher G (2003). Cerebral and peripheral effects of laserneedle-stimulation. *Neurological Research*.

[21] Litscher G, Huang T, Wang L, Zhang W (2010). Violet laser acupuncture-part 1: effects on brain circulation. *Journal of Acupuncture and Meridian Studies*.

[22] Wang L, Huang T, Zhang W, Litscher G (2011). Violet laser acupuncture-part 2: effects on peripheral microcirculation. *Journal of Acupuncture and Meridian Studies*.

[23] Litscher G, Wang L, Huang T, Zhang W (2011). Violet laser acupuncture—part 3: a pilot study on the potential effects on temperature distribution. *Journal of Acupuncture and Meridian Studies*.

[24] Litscher G, Wang L, Gaischek I, Gao X-Y (2011). Violet laser acupuncture-part 4: acute effects on human arterial stiffness and wave reflection. *Journal of Acupuncture and Meridian Studies*.

[25] Litscher G, Xie Z, Wang L, Gaischek I (2009). Blue 405 nm laser light mediates heart rate—investigations at the acupoint Neiguan (Pe.6) in Chinese adults. *North American Journal of Medical Sciences*.

[26] Byrnes KR, Waynant RW, Ilev IK (2005). Light promotes regeneration and functional recovery and alters the immune response after spinal cord injury. *Lasers in Surgery and Medicine*.

[27] Cayce JM, Friedman RM, Jansen ED, Mahavaden-Jansen A, Roe AW (2011). Pulsed infrared light alters neural activity in rat somatosensory cortex in vivo. *NeuroImage*.

[28] Chuang CM, Hsieh CL, Li TC, Lin JG (2007). Acupuncture stimulation at Baihui acupoint reduced cerebral infarct and increased dopamine levels in chronic cerebral hypoperfusion and ischemia-reperfusion injured Sprague-Dawley rats. *American Journal of Chinese Medicine*.

[29] Baxter GD, Bleakley C, McDonough S (2008). Clinical effectiveness of laser acupuncture: a systematic review. *Journal of Acupuncture and Meridian Studies*.

